# Transferring robotic expertise from multi‐port to single‐port partial nephrectomy: A comparative analysis of perioperative outcomes and learning curves

**DOI:** 10.1002/bco2.70253

**Published:** 2026-07-12

**Authors:** Andres Affentranger, Silvan Sigg, David Abt, Miranda Fanconi, Christoph Würnschimmel, Philipp Baumeister, Agostino Mattei, Christian D. Fankhauser, Ernest Kaufmann

**Affiliations:** ^1^ Department of Urology University Teaching and Research Hospital University of Lucerne Lucerne Switzerland; ^2^ Faculty of Health Sciences and Medicine University of Lucerne Lucerne Switzerland; ^3^ Faculty of Medicine University of Zurich Zurich Switzerland; ^4^ Division of Urology, Department of Surgery Cantonal Hospital of Winterthur Winterthur Switzerland

**Keywords:** learning curve, perioperative outcome, robot‐assisted partial nephrectomy, single port

## Abstract

**Objective:**

The objective of this study is to evaluate perioperative outcomes and learning curves following implementation of single‐port robot‐assisted partial nephrectomy (SP‐RAPN) at a European tertiary referral centre and to assess the transferability of robotic expertise from established multi‐port (MP) platforms to single‐port (SP) surgery.

**Patients and Methods:**

This single‐centre retrospective cohort study included consecutive patients undergoing RAPN between February 2023 and August 2025. Procedures were performed by four surgeons (two experts and two trainees) using da Vinci Xi MP or da Vinci SP systems. Primary outcomes included operative time, warm ischemia time, complications and trifecta (negative margins, ischemia ≤25 min and no major complications). Learning curves were analyzed using risk‐adjusted CUSUM methodology.

**Results:**

MP‐RAPN was performed in 65 patients and SP‐RAPN in 62. Mean operative time was 193 min in both groups, and trifecta was 80% for MP‐RAPN versus 69% for SP‐RAPN (*p* = 0.17); however, given the limited sample size, we could not rule out a clinically meaningful difference. Intraoperative complications occurred in three MP‐patients (5%) and in no SP‐patients. Length of stay was significantly shorter following SP‐RAPN (2 vs. 3 days, *p* < 0.001). CUSUM analysis demonstrated a potential learning curve in experienced surgeons (*R* ^2^ = 0.91, proficiency at case 18), with patterns in other surgeons limited by case volume.

**Conclusion:**

SP‐RAPN can be safely implemented in experienced robotic centres with outcomes comparable to MP approaches and shorter hospital stays. Excellent skill transferability from MP to SP platforms was demonstrated in experienced surgeons. Supervised trainee adoption was feasible without complications.

## 1 INTRODUCTION

Robot‐assisted partial nephrectomy (RAPN) has emerged as the preferred surgical approach for the treatment of localized renal cell carcinoma (RCC).^1^ Traditionally, Multi‐Port RAPN (MP‐RAPN) has been performed via transperitoneal access with the patient in lateral flank position, a time‐consuming setup associated with increased postoperative pain, pressure‐related complications and the need for bowel mobilization, carrying additional risks of bowel injury, postoperative ileus, anaesthetic complications related to pneumoperitoneum and technical challenges in patients with prior abdominal surgery.^2^, ^3^, ^4^, ^5^ In contrast, retroperitoneal RAPN avoids intraperitoneal manipulation, but its widespread adoption has been hindered by technical complexity and frequent instrument collisions in the confined retroperitoneal space, often still requiring lateral flank positioning.^6^ Single‐port (SP) robotic systems have revitalized interest in the retroperitoneal approach by addressing both limitations: SP platforms are designed for narrow anatomical spaces, minimizing instrument conflicts and reducing inadvertent pneumoperitoneum, while enabling procedures in supine position.^7^, ^8^, ^9^, ^10^

Early clinical experience and multicentre prospective studies have demonstrated technical feasibility, low complication rates, reduced blood loss and shorter hospital stay.^7^, ^8^, ^11^, ^12^, ^13^ However, European data on SP‐RAPN outcomes and learning curves remain limited, and understanding how surgeons with varying robotic experience adapt to SP systems is critical for safe implementation.^14^ We therefore compared perioperative outcomes between MP‐RAPN and SP‐RAPN and characterized the learning curve following SP system introduction at a single European centre using risk‐adjusted CUSUM analysis, stratified by surgeon experience level.

## 2 METHODS

### 2.1 Study design and setting

This single‐centre retrospective observational cohort study at a tertiary referral centre included all consecutive patients who underwent RAPN between February 2023 and August 2025. The study was conducted in accordance with the Declaration of Helsinki, the Guidelines on Good Clinical Practice issued by the European Medicines Agency, Swiss law and regulatory authority requirements and the Strengthening the Reporting of Observational Studies in Epidemiology (STROBE) guidelines. Ethical approval was obtained from the local ethics committee (Approval Number 2020‐02389).

### 2.2 Population

All consecutive patients undergoing RAPN for localized renal masses during the study period were included. Patients younger than 18 years and those without written general consent were excluded. Procedures were performed by four surgeons with varying levels of robotic surgical experience. Two senior attending surgeons with extensive prior MP robotic experience (both >1000 procedures) were designated as Experts 1 and 2. Two urology consultants in their third year of robotic training were designated as Trainees 1 and 2. MP‐RAPN was performed using the Da Vinci Xi® system (Intuitive Surgical, Sunnyvale, CA, United States) throughout the study period, and SP‐RAPN was performed using the Da Vinci SP® system, which was introduced at our institution in May 2024.

### 2.3 Surgical procedure

The choice of surgical approach and robotic platform was determined by the operating surgeon based on tumour location, patient anatomy, system availability and prior abdominal surgery.

All MP procedures were performed using transperitoneal access, with patients positioned in flank position. For SP procedures, retroperitoneal access was preferentially employed using one of three techniques based on tumour location: low anterior retroperitoneal access (LARA), supine subcostal access (SUSA) or lateral flank access (LFA). Patients undergoing SP procedures were positioned in supine or modified lateral position. Detailed descriptions of access techniques and patient positioning protocols have been published previously.^15^ Details on the institutional robot‐assisted partial nephrectomy technique have been described in detail elsewhere.^16^ For SP procedures, the following technical modifications were implemented to accommodate the SP platform: First, an additional assistant trocar (+1) was routinely placed to facilitate bulldog clamp positioning. Second, endoscopic specimen retrieval bags were utilized selectively for cystic lesions only, as solid lesions could be removed through the floating dock. Third, owing to restricted lateral reach within the constrained working envelope of the SP system, renorrhaphy sutures were passed sequentially during reconstruction rather than being preplaced prior to tumour excision. Fourth, fibrin sealant (Tisseel, 4 ml [Baxter Healthcare Corp., Deerfield, IL]) was applied to the resection bed using a flexible instead of a rigid tip applicator to achieve optimal angulation for haemostasis before initiating renorrhaphy.

### 2.4 Data collection and perioperative outcomes

Patient demographics, preoperative characteristics, tumour features, operative details and perioperative outcomes were prospectively recorded in a dedicated institutional database and retrospectively analysed. Intraoperative variables included operative time (skin incision to skin closure), warm ischemia time, estimated blood loss, conversion to radical nephrectomy and intraoperative complications graded according to ClassIntra.^17^ Postoperative outcomes included length of hospital stay, postoperative complications graded according to the Clavien‐Dindo classification, Comprehensive Complication Index (CCI) and final histopathology.^18^, ^19^ Trifecta achievement was defined as negative surgical margins, warm ischemia time ≤25 min, and absence of perioperative complications ≥Clavien‐Dindo Grade 3.

### 2.5 Learning curves analysis

Learning curves were analyzed using risk‐adjusted cumulative sum (CUSUM) analysis for operative time.^20^ For each surgeon and experience level (expert vs. trainee), procedures were ordered chronologically, and the mean operative time (*μ*) was calculated as the reference value. The CUSUM value for each case was computed using the following formula:

$${\text{CUSUM}}_{n}={\text{CUSUM}}_{n-1}+\left(X_{n}-\mu \right),$$

where *μ* represents the surgeon‐specific mean operative time, *X*_*n*_ the operative time for case *n* and *CUSUM*_*n*_ the cumulative sum for case *n*. The initial CUSUM value (*CUSUM*₁) was set to zero. CUSUM curves were constructed by plotting cumulative values on the *y* axis against sequential case numbers on the *x* axis. To characterize the nonlinear trend in the data, a cubic polynomial regression (Degree 3) was performed.^13^, ^20^ The goodness of fit for each model was assessed using the coefficient of determination (*R* ^2^) with values closer to 1 indicating a stronger fit. The resulting CUSUM curve enabled evaluation of trends in operative performance. An upward slope reflects the learning phase, where operative times consistently exceed the surgeon‐specific mean and a downward slope indicates progressive improvement, with operative times falling below the mean. The learning curve therefore displays an inverted U‐shaped pattern: an initial ascending phase during learning, followed by a descending phase as proficiency is achieved. The inflection point, identified as the maximum of the curve, represents the transition from learning to proficiency. A stable pattern around the zero line denotes consistent performance at the mean level. Overall, this approach offers an objective means of assessing surgical efficiency over time.

### 2.6 Statistical analysis

Continuous variables were assessed for normality using the Shapiro–Wilk test. Normally distributed variables are presented as mean ± standard deviation and compared using Student's *t* test or ANOVA with Tukey's post hoc test. Non‐normally distributed variables are presented as median (IQR) and compared using Mann–Whitney *U* or Kruskal–Wallis tests. Categorical variables are expressed as frequencies and percentages and compared using chi‐square or Fisher's exact test. Comparative analyses between MP and SP groups were performed for baseline characteristics, operative details and perioperative outcomes, stratified by experience level and individual surgeon. For surgeons with well‐defined learning curves based on CUSUM analysis, outcomes were compared between cases before and after the inflection point to assess the impact of achieving proficiency.

All statistical tests were two‐sided, and a *p* value of <0.05 was considered statistically significant. Statistical analyses were performed using R Version 4.5.1 (R Foundation for Statistical Computing, Vienna, Austria).

## 3 RESULTS

### 3.1 Study cohort characteristics

A total of 127 patients underwent RAPN between February 2023 and August 2025. Most patients were male (72%, *n* = 92) with a mean age of 62 years (SD 11). Trifecta was achieved in 75% of cases (*n* = 90). Failure to achieve trifecta was attributed to warm ischemia time exceeding 25 min in 21% of patients (*n* = 32), positive surgical margins in 2% (*n* = 2) and postoperative complications ≥Clavien‐Dindo Grade 3a in 4% (*n* = 5). Detailed patient characteristics and perioperative outcomes are presented in Tables 1 and S1.

**TABLE 1:** Key patient characteristics and outcomes stratified by robotic platform.

Characteristic	Overall *N* = 127	Robot‐assisted multi‐port *N* = 65	Robot‐assisted single‐port *N* = 62	*p* value
Age, years	62 (11)	62 (9)	62 (13)	0.7
Gender				0.12
Female	35 (28%)	14 (22%)	21 (34%)	
Male	92 (72%)	51 (78%)	41 (66%)	
BMI, kg/m^2^	28.6 (5.2)	28.9 (5.6)	28.3 (4.9)	0.9
Charlson Comorbidity Index	2 (2, 4)	2 (2, 3)	2 (2, 4)	0.6
ASA Classification				0.2
1	2 (2%)	1 (2%)	1 (2%)	
2	63 (50%)	32 (49%)	31 (50%)	
3	58 (46%)	32 (49%)	26 (42%)	
4	4 (3%)	0 (0%)	4 (7%)	
Anticoagulation	40 (31%)	21 (32%)	19 (31%)	0.8
Preoperative eGFR, ml/min/1.73 m^2^	80 (64, 90)	78 (62, 86)	87 (71, 90)	0.017
Imaging modality				0.2
CT	84 (66%)	48 (74%)	36 (58%)	
CT and MRI	25 (20%)	10 (15%)	15 (24%)	
MRI	17 (13%)	7 (11%)	10 (16%)	
Tumour size, mm	32 (23, 49)	32 (25, 49)	32 (22, 49)	0.8
Lesion type				>0.9
Suspected cancer	106 (83%)	55 (85%)	51 (82%)	
Cystic lesion (Bosniak Cyst ≥ 2F)	8 (6%)	4 (6%)	4 (6%)	
Suspected benign tumour	13 (10%)	6 (9.2%)	7 (11%)	
T Stage				>0.9
cT1a	67 (53%)	34 (52%)	33 (53%)	
cT1b	27 (21%)	13 (20%)	14 (23%)	
cT2a	6 (5%)	4 (6%)	2 (3%)	
cT2b	2 (2%)	1 (2%)	1 (2%)	
cT3a	4 (3%)	2 (3%)	2 (3%)	
Not applicable/unknown	21 (17%)	11 (17%)	10 (16%)	
N stage				0.6
cN0	125 (98%)	63 (97%)	62 (100%)	
cN1	2 (2%)	2 (3%)	0 (0%)	
M stage				0.6
cM0	127 (100%)	65 (100%)	62 (100%)	
Bosniak classification				0.2
2F	1 (1%)	1 (2%)	0 (0%)	
3	4 (3%)	3 (5%)	1 (2%)	
4	3 (2%)	0 (0%)	3 (5%)	
Surgical approach				<0.001
Retroperitoneal	31 (24%)	0 (0%)	31 (50%)	
Transperitoneal	96 (76%)	65 (100%)	31 (50%)	
Access technique				<0.001
Lateral flank access (retroperitoneal)	13 (10%)	0 (0%)	13 (21%)	
Low anterior retroperitoneal access (LARA)	9 (7%)	0 (0%)	9 (15%)	
Supine subcostal retroperitoneal access (SUSA)	9 (7%)	0 (0%)	9 (15%)	
Transperitoneal access	96 (76%)	65 (100%)	31 (50%)	
Surgery time, min	193 (54)	193 (48)	193 (59)	0.9
Ischemia time, min	21 (16, 25)	18 (14, 24)	21 (17, 30)	0.059
Intraoperative blood loss, ml	100 (50, 200)	200 (100, 300)	90 (50, 175)	0.008
Trifecta reached	90 (75%)	49 (80%)	41 (69%)	0.2
Ischemia time ≤20 min	95 (79%)	52 (85%)	43 (73%)	0.1
Negative resection margin	125 (98%)	64 (98%)	61 (98%)	0.9
No complication ≥CDC Grade 3a	122 (96%)	61 (94%)	61 (98%)	0.4
Conversion				0.5
Conversion to nephrectomy	1 (1%)	0 (0%)	1 (2%)	
Intraoperative complication	3 (2%)	3 (5%)	0 (0%)	0.2
Type of intraoperative complication				0.9
Colon perforation	1 (1%)	1 (2%)	0 (0%)	
Small bowel perforation	1 (1%)	1 (2%)	0 (0%)	
Injury of renal vein	1 (1%)	1 (2%)	0 (0%)	
ClassIntra grade of intraoperative complication				0.2
Grade 2	3 (2%)	3 (5%)	0 (0%)	
Comprehensive Complication index	21 (21, 39)	21 (21, 42)	0 (0, 34)	0.2
Length of stay	2 (2, 3)	3 (2, 3)	2 (2, 2)	<0.001
Pathology				0.8
Clear cell RCC	60 (47%)	31 (48%)	29 (47%)	
Papillary RCC	23 (18%)	11 (17%)	12 (19%)	
Chromophobe RCC	10 (8%)	6 (9%)	4 (7%)	
Other malignant	7 (6%)	2 (3%)	5 (8%)	
Oncocytoma	16 (13%)	8 (13%)	8 (13%)	
Angiomyolipoma	5 (4%)	4 (6%)	1 (2%)	
Other benign	6 (4%)	3 (4%)	3 (4%)	

*Note*: Mean (SD) for normally distributed continuous variables; median (Q1 and Q3) for nonnormally distributed continuous variables; *n* (%) for categorical variables. Group comparisons performed using Student's *t* test for normally distributed continuous variables, Wilcoxon rank‐sum test for nonnormally distributed continuous variables and Pearson's chi‐squared test or Fisher's exact test for categorical variables.

Abbreviations: ASA, American Society of Anesthesiologists; BMI, Body Mass Index; CDC, Clavien–Dindo Classification; eGFR, estimated glomerular filtration rate; *N*, number; RCC, renal cell carcinoma.

### 3.2 Robotic platform and surgical access

MP‐RAPN was performed in 51% of patients (*n* = 65), exclusively via transperitoneal access. Following introduction of the single‐port (SP) system in May 2024, the proportion of MP‐RAPN procedures decreased to 13% (9 of 71 subsequent cases).

**FIGURE 1:**
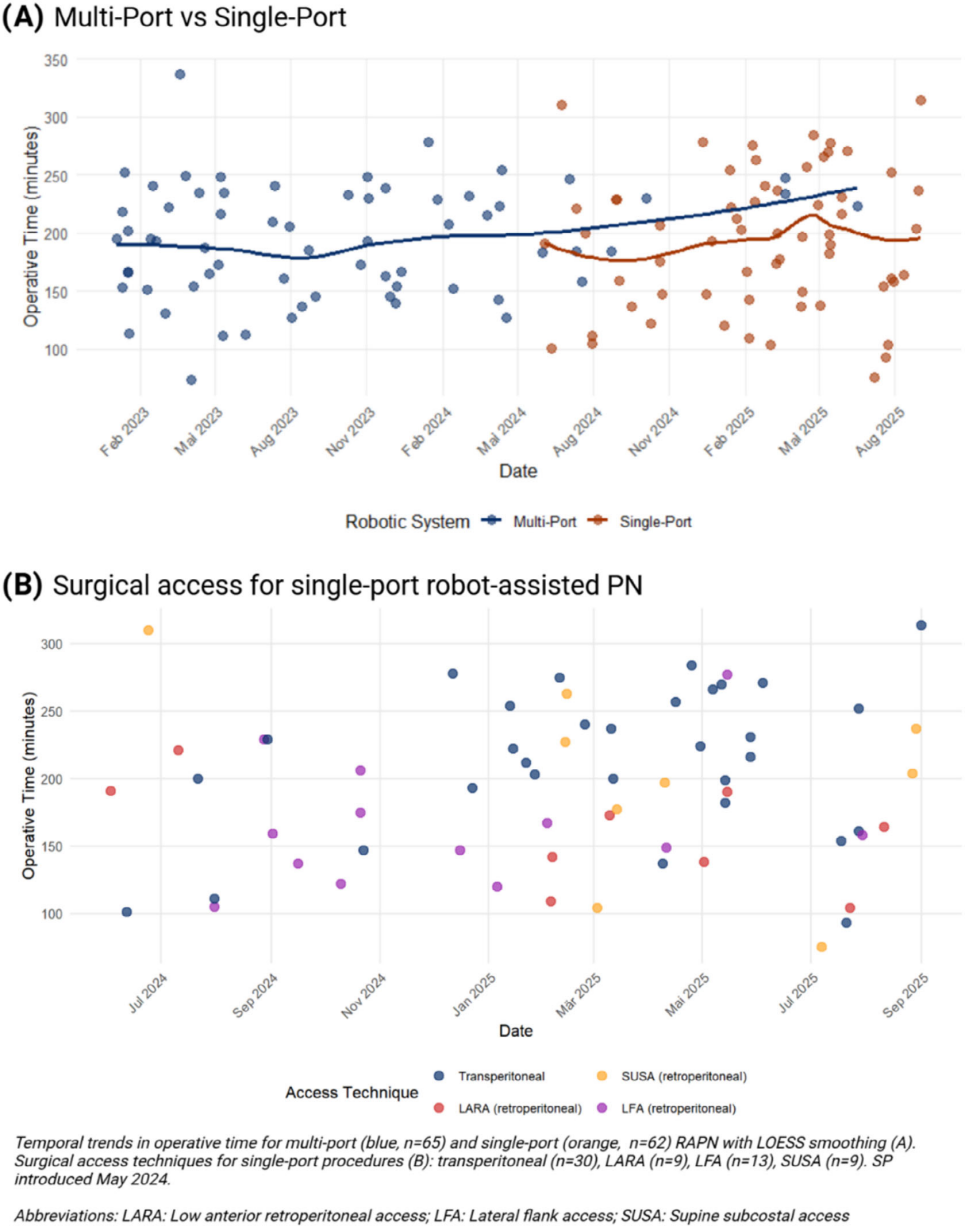
Temporal trends in operative time for RAPN stratified by robotic platform and surgical approach. (A) Operative time trends for multi‐port (blue, *n* = 65) and single‐port (orange, *n* = 62) RAPN over time with LOESS trend lines. The SP robotic platform was introduced in May 2024. (B) Operative time trends for SP‐RAPN stratified according to surgical access technique, including transperitoneal access (blue, *n* = 31), low anterior retroperitoneal access (LARA, red, *n* = 9), lateral flank access (LFA, purple, *n* = 13) and supine subcostal access (SUSA, yellow, *n* = 9). Abbreviations: LARA, low anterior retroperitoneal access; LFA, lateral flank access; LOESS, locally estimated scatterplot smoothing; MP, multi‐port; RAPN, robot‐assisted partial nephrectomy; SP, single‐port; SUSA, supine subcostal access.

Among SP‐RAPN procedures, half were performed via transperitoneal access (50%, *n* = 31). The most frequently used retroperitoneal approaches were LFA (21%, *n* = 13), LARA (15%, *n* = 9) and SUSA (15%, *n* = 9). Temporal trends in platform utilization and access route selection are illustrated in Figure 1. Comparative outcomes between transperitoneal and retroperitoneal SP‐RAPN have been previously published^21^ and are summarized in Table S2.

### 3.3 MP versus SP robotic surgery

Baseline patient characteristics were comparable between MP‐RAPN and SP‐RAPN groups, with the exception of preoperative eGFR, which was higher in the SP‐RAPN cohort (87 vs. 78 ml/min/1.73m^2^, *p* = 0.02). Median tumour size (32 mm in both groups) and mean operative time (193 min in both groups) did not differ between platforms. However, median warm ischemia time tended to be longer in SP‐RAPN (18 vs. 21 min, *p* = 0.059).

Intraoperative complications occurred in three MP‐RAPN patients (5%), all classified as ClassIntra Grade 2: one colon injury during port insertion requiring conversion to open surgery (uneventful recovery, hospital discharge on postoperative Day 2), one small bowel perforation managed with robotic suturing and one renal vein injury repaired robotically. No intraoperative complications were observed in the SP‐RAPN cohort. Trifecta achievement rates (MP‐RAPN 80% [*n* = 49] vs. SP‐RAPN 69% [*n* = 41]), ischemia time exceeding 25 min (MP‐RAPN 15% [*n* = 13] vs. SP‐RAPN 27% [*n* = 19]), positive surgical margins (MP‐RAPN 2% [*n* = 1] vs. SP‐RAPN 2% [*n* = 1]) and major postoperative complications (MP‐RAPN 6% [*n* = 4] vs. SP‐RAPN 2% [*n* = 1]) showed no significant differences. Although some endpoints favoured MP‐RAPN numerically and others favoured SP‐RAPN, the limited sample size precludes definitive conclusions about clinically meaningful differences (all *p* > 0.05).

Major postoperative complications (≥Clavien‐Dindo Grade 3a) included bleeding from renal pseudoaneurysm requiring coiling in one MP‐RAPN and one SP‐RAPN patient, urinoma requiring ureteral stent placement in two MP‐RAPN patients, and seroma requiring drainage in one MP‐RAPN patient. Median length of hospital stay was significantly shorter following SP‐RAPN compared to MP‐RAPN (MP‐RAPN 3 days [IQR 2–3] vs. SP‐RAPN 2 days [IQR 2–3], *p* < 0.001).

### 3.4 CUSUM learning curve analysis

**FIGURE 2:**
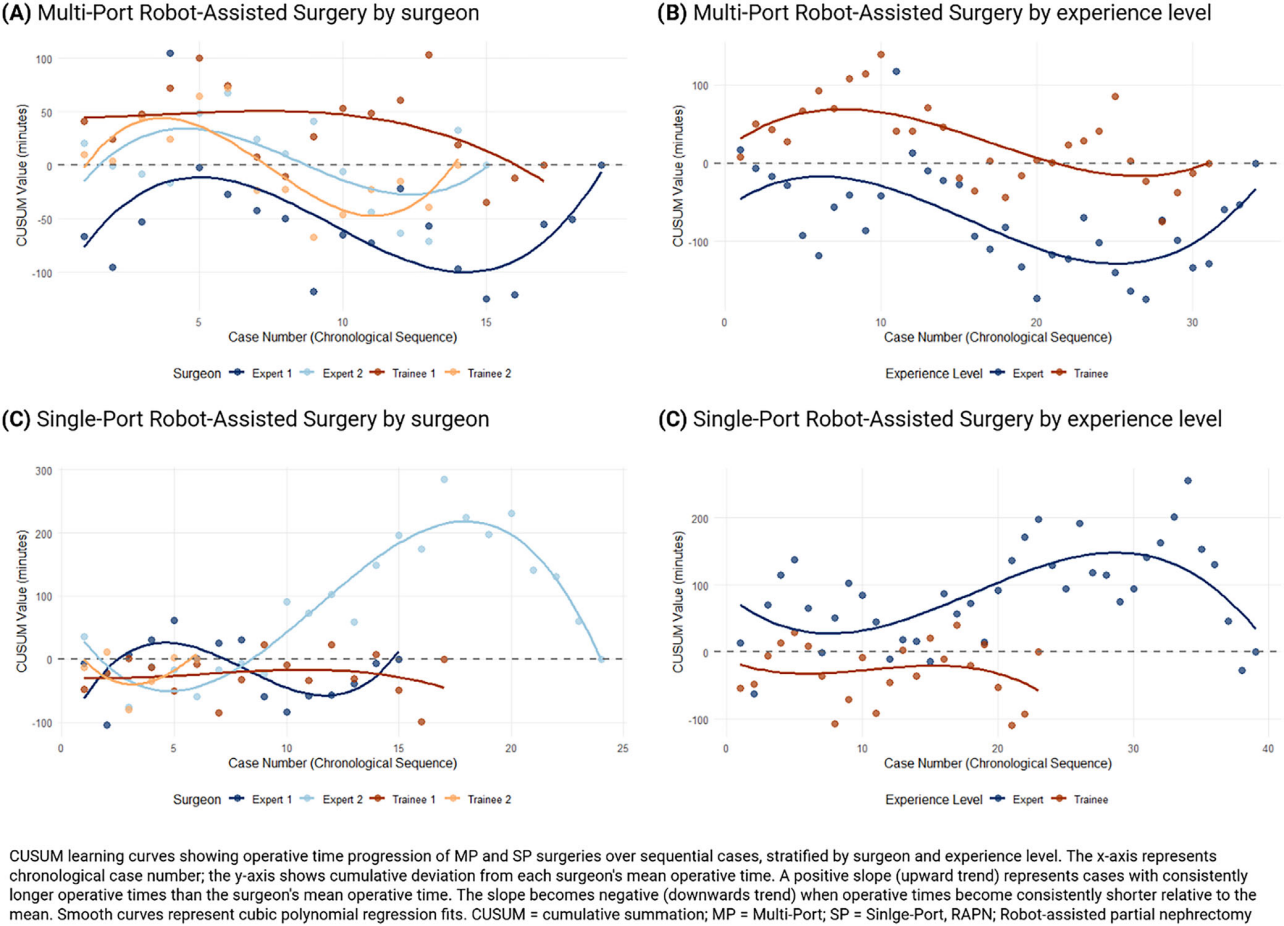
CUSUM learning curves for operative time in multi‐port and single‐port RAPN stratified by surgeon and experience level. (A) CUSUM curves for MP‐RAPN stratified by individual surgeon. (B) CUSUM curves for MP‐RAPN stratified by surgeon experience level (expert vs. trainee). (C) CUSUM curves for SP‐RAPN stratified by individual surgeon. (D) CUSUM curves for SP‐RAPN stratified by surgeon experience level (expert vs. trainee). The *x* axis represents chronological case sequence and the *y* axis cumulative deviation from the surgeon‐specific mean operative time. Upward slopes indicate operative times above the surgeon‐specific mean (learning phase), whereas downward slopes indicate operative times below the mean (proficiency phase). Smooth curves represent cubic polynomial regression fits. Abbreviations: CUSUM, cumulative sum; MP, multi‐port; RAPN, robot‐assisted partial nephrectomy; SP, single‐port.

The learning curves for operation time of MP and SP‐RAPN stratified by surgeon and experience level are shown in Figure 2. After CUSUM calculation and learning curve construction, MP‐RAPN demonstrated no clear model fit across all surgeons and experience levels (*R* ^2^ range: 0.25–0.63), with late inflection points suggesting prior proficiency with this established platform. In contrast, SP‐RAPN revealed distinct learning patterns. Pooled expert analysis yielded proficiency at case 29 (*R* ^2^ = 0.37), whereas trainee data showed lower model fit (*R* ^2^ = 0.04). Individual surgeon analysis demonstrated that Expert 2 had the most pronounced learning curve with good model fit (*R* ^2^ = 0.91), achieving proficiency at Case 18 as evidenced by the transition from ascending (learning phase) to descending (proficiency phase) CUSUM values. Expert 1 showed a less pronounced but stable learning curve, reaching proficiency at case 12 (*R* ^2^ = 0.44). Temporal trends in operative time and ischemia time without CUSUM calculation, using smoothed trend lines and linear regression, are presented in Figures S1–S4 and risk of not achieving trifecta over consecutive cases is shown in Figure S5.

### 3.5 Comparative analysis of learning and proficiency phase

To assess the impact of achieving proficiency on perioperative outcomes, Expert 2's single‐port cases were divided into learning phase (Cases 1–18) and proficiency phase (Cases 19–24) based on the CUSUM‐derived inflection point. Patient characteristics were comparable between phases. Tumour characteristics were also similar, with median tumour size 40 mm (IQR 21–54) in the learning phase versus 35 mm (IQR 25–49) in the proficiency phase (*p* = 0.7). Patient characteristics and perioperative outcomes for Expert 2 are summarized in Table S3.

Mean operative time decreased from 177 min (SD 57) during the learning phase to 127 min (SD 45) after achieving proficiency (*p* = 0.053). Median warm ischemia time remained stable at 20 min (IQR 16–24) in both phases (*p* = 0.9). Trifecta achievement rates were comparable between phases: 78% (*n* = 14/18) during learning versus 83% (*n* = 5/6) after proficiency (*p* > 0.9). Failure to achieve trifecta was attributed exclusively to warm ischemia time exceeding 25 min in 22% (*n* = 4/18) during learning and 17% (*n* = 1/6) after proficiency. No intraoperative complications, postoperative complications ≥Clavien‐Dindo Grade 3a or positive surgical margins occurred in either phase.

## 4 DISCUSSION

This study evaluates the transition from MP to SP‐RAPN in a real‐world European setting, with focus on perioperative outcomes and surgeon‐specific learning curves. Our findings demonstrate that SP‐RAPN can be safely implemented in experienced robotic centres without compromising surgical outcomes, while offering potential advantages in length of hospital stay. The results provide insight into the transferability of robotic expertise and feasibility of SP adoption across different experience levels.

A key finding is the comparable safety profile between platforms. No intraoperative complications occurred in the SP cohort, whereas three intraoperative adverse events were observed in the MP group, including bowel injuries and renal vein injury. Although absolute numbers are small, this observation aligns with proposed advantages of retroperitoneal SP approaches in avoiding intraperitoneal organ manipulation and reducing bowel‐related complications.^22^

Postoperative outcomes were comparable between platforms, with no significant differences in major complications (MP 6% vs. SP 2%, *p* > 0.05), positive surgical margins (MP 2% vs. SP 2%) or trifecta achievement (MP 80% vs. SP 69%, *p* > 0.05). These findings are consistent with contemporary SP‐RAPN series reporting low morbidity and preserved oncologic quality, supporting the non‐inferiority of SP relative to MP approaches.^23^

SP‐RAPN was associated with a significantly shorter length of hospital stay (2 days [IQR 2–3] vs. 3 days [IQR 2–3], *p* < 0.001). Although modest in absolute terms, even a 1‐day reduction has meaningful implications for healthcare resource utilization, given daily inpatient costs exceeding $1000–$3000 in many healthcare systems. Similar reductions in hospitalization have been reported in early SP‐RAPN experiences, where rapid recovery, low postoperative pain scores and early discharge were observed, corroborating growing evidence that SP approaches may facilitate enhanced recovery pathways.^23^, ^24^

Understanding the learning curve associated with SP‐RAPN adoption is essential for safe implementation. In our cohort, learning curve patterns varied substantially between surgeons. A well‐defined CUSUM curve (*R* ^2^ = 0.91) was observed exclusively for Expert 2, who achieved proficiency after 18 cases with a 50‐min reduction in mean operative time (177–127 min, *p* = 0.053). Other surgeons demonstrated variable or poorly fitting curves (*R* ^2^ = 0.05–0.44), likely reflecting limited case numbers that can obscure true performance trends and delay identification of proficiency thresholds.

Learning curves are likely to differ substantially between surgeons, particularly between trainees and those with an established MP robotic background. In our experience, SP‐RAPN adds a further layer of technical complexity beyond MP‐RAPN, driven by the required precision in access, a more constrained working space, modified instrument handling and the need to adapt dissection technique to smaller instruments generating less force for tissue and plane dissection. Consequently, SP adoption should be reserved for centres with sufficient case volume to support a meaningful learning curve. Transitioning to SP‐RAPN without adequate institutional volume risks an incomplete learning curve and, importantly, may compromise patient safety during the adoption phase.

Importantly, our results indicate that SP adoption is feasible not only for expert surgeons but also for trainees. Although no consistent learning curves were observed among trainees, primarily due to limited case numbers, no intraoperative or major postoperative complications occurred in trainee‐performed SP procedures. All trainee cases were performed under close intraoperative supervision by experienced robotic surgeons, with structured stepwise teaching and real‐time guidance. This supervised model likely mitigated risks during early adoption and supports the safe integration of SP‐RAPN into training programs. Nevertheless, it is reasonable to assume that trainees require a longer learning curve, which could not be reliably quantified in our cohort due to limited procedural volume.

Recent literature provides important context and aligns with our findings. Santarelli et al.^6^ reported that approximately 33 cases were required for an experienced robotic surgeon to achieve proficiency in SP‐RAPN via LARA access, with improvements in operative time, ischemia time, complication rates and trifecta achievement. Similarly, van Eecke et al.^24^ demonstrated that SP‐RAPN via LARA can be safely adopted by a SP‐naive surgeon with extensive MP experience, showing low complication rates, minimal pain, and early discharge despite the early learning phase.

However, in contrast to these high‐volume or single‐surgeon experiences, our multi‐surgeon setting with lower case numbers did not demonstrate a uniform proficiency threshold. Although trends toward improved operative efficiency were observed, they did not reach statistical significance, likely reflecting limited sample size and heterogeneity in surgeon experience. These findings suggest that although SP‐RAPN can be safely adopted early, the number of cases required to achieve measurable efficiency gains may vary substantially depending on surgical volume, supervision and prior robotic experience.

Our study is subject to several limitations inherent to its design. First, as a single‐centre retrospective observational study, the choice of surgical platform and access route was determined by the operating surgeon, introducing potential selection bias. Although baseline characteristics were comparable between groups, unmeasured confounders may have influenced perioperative outcomes. Second, the moderate overall sample size and limited number of cases per surgeon restrict statistical power and generalizability, particularly for learning curve analyses. The distribution of cases across multiple surgeons with varying levels of experience further complicates interpretation of proficiency thresholds, as cumulative analyses may dilute individual learning effects. Third, SP adoption coincided with ongoing training and evolving case complexity, making it difficult to isolate the independent impact of the new platform on operative efficiency and outcomes. Fourth, the absence of standardized tumour complexity scoring, such as the RENAL nephrometry score, limits comparability with existing literature and may have influenced platform selection. Finally, the present analysis focuses on short‐term perioperative outcomes and lacks long‐term oncologic and functional follow‐up, which are essential to fully assess the durability and clinical effectiveness of SP‐RAPN.

## 5 CONCLUSION

SP‐RAPN can be safely implemented in centres with established multi‐port robotic expertise, providing perioperative outcomes comparable to the current standard while reducing length of hospital stay. Our findings, consistent with contemporary SP‐RAPN series, support the transferability of robotic skills and the feasibility of supervised trainee adoption.

## AUTHOR CONTRIBUTIONS

Christian Daniel Fankhauser had full access to all the data in the study and takes responsibility for the integrity of the data and the accuracy of the data analysis. *Study concept and design*: Ernest Kaufmann and Christian D. Fankhauser. *Acquisition of data*: David Abt, Miranda Fanconi, Ernest Kaufmann and Andres Affentranger. *Analysis and interpretation of data*: Andres Affentranger, Ernest Kaufmann and Christian D. Fankhauser. *Drafting of the manuscript*: Andres Affentranger, Ernest Kaufmann and Christian D. Fankhauser. *Critical revision of the manuscript for important intellectual content*: All Authors. *Statistical analysis*: Andres Affentranger, Ernest Kaufmann and Christian D. Fankhauser. *Obtaining funding*: None. *Administrative, technical or material support*: Christian D. Fankhauser, Agostino Mattei, Christoph Würnschimmel and Philipp Baumeister. *Supervision*: Ernest Kaufmann and Christian D. Fankhauser. *Other*: None.

## CONFLICT OF INTEREST STATEMENT

The authors declare no conflicts of interest.

## Supporting information


Supporting Information S1.

## Data Availability

The data that support the findings of this study are available from the corresponding author upon reasonable request.
