# Supplementary material for: Transferring robotic expertise from multi‐port to single‐port partial nephrectomy: A comparative analysis of perioperative outcomes and learning curves
Source: BJUI Compass. 2026 Jul 12;7(7):e70253. doi: 10.1002/bco2.70253 (PMC13357013; doi:10.1002/bco2.70253)
Supplement: Supplementary file 1 — Supporting Information S1. [file BCO2-7-e70253-s001.pdf]

Supplementary Figures

Supplementary Figure 1: Operation time of MP and SP surgeries stratified by surgeon and experience level

A) Multi-port robot-assisted surgery by surgeon

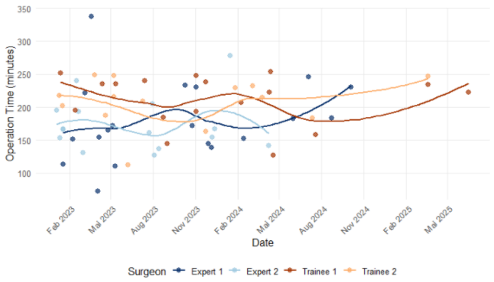

B) Multi-port robot-assisted surgery by experience level

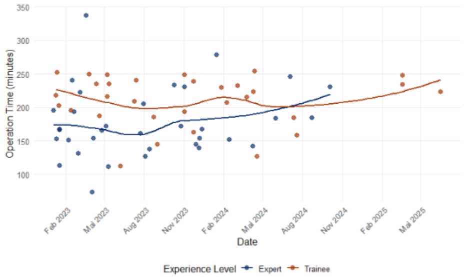

C) Single-port robot-assisted surgery by surgeon

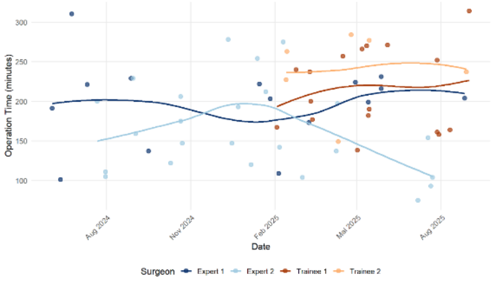

C) Single-port robot-assisted surgery by experience level

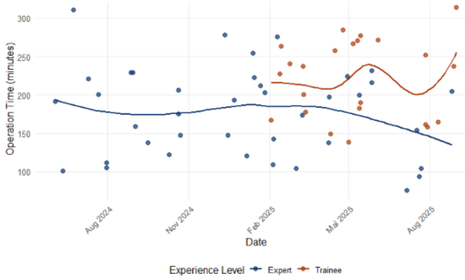

Temporal trends in operative time for multi-port (A-B) and single-port (C-D) robot-assisted partial nephrectomy. Individual surgeons (A, C) and pooled experience levels (B, D) are shown. Trend lines fitted using locally weighted scatterplot smoothing (LOESS). For surgeons with ≥10 cases, span=0.75; for Trainees with <10 cases, span=2.0 to prevent overfitting.

Supplementary Figure 2: Operation time of MP and SP surgeries stratified by surgeon and experience level

A) Multi-port robot-assisted surgery by surgeon

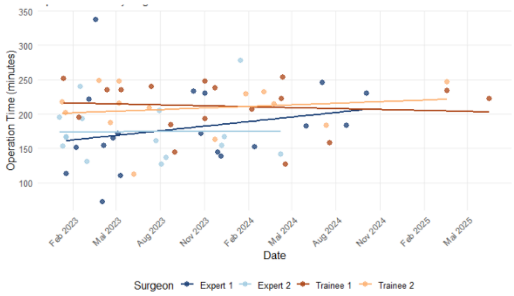

B) Multi-port robot-assisted surgery by experience level

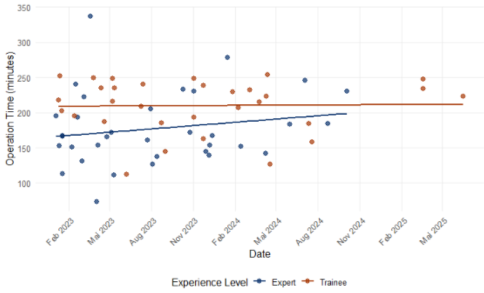

C) Single-port robot-assisted surgery by surgeon

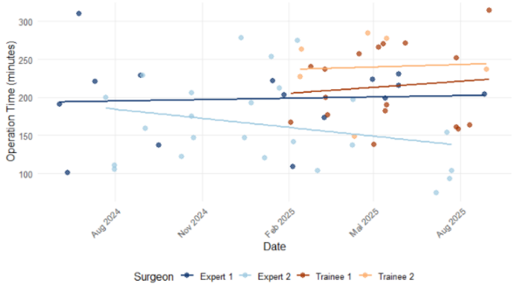

C) Single-port robot-assisted surgery by experience level

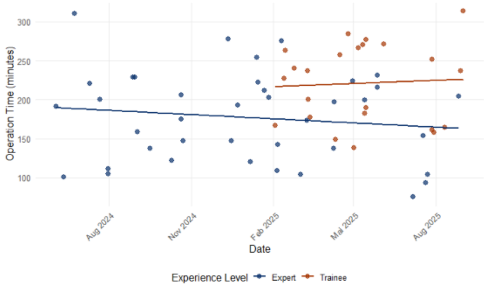

Temporal trends in operative time for multi-port (A-B) and single-port (C-D) robot-assisted partial nephrectomy. Individual surgeons (A, C) and pooled experience levels (B, D) are shown. Trend lines fitted using linear regression.

Supplementary Figure 3: Ischemia time of MP and SP surgeries stratified by surgeon and experience level

A) Multi-port robot-assisted surgery by surgeon

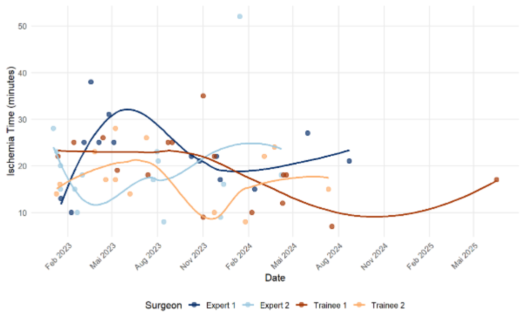

B) Multi-port robot-assisted surgery by experience level

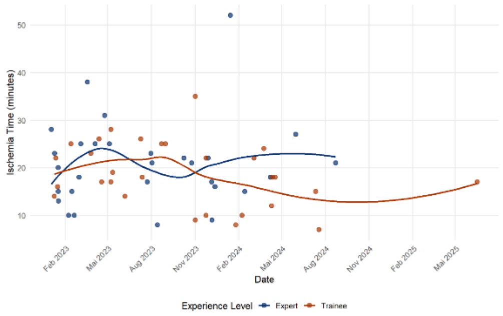

C) Single-port robot-assisted surgery by surgeon

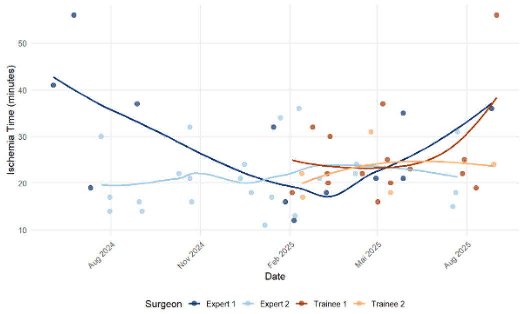

C) Single-port robot-assisted surgery by experience level

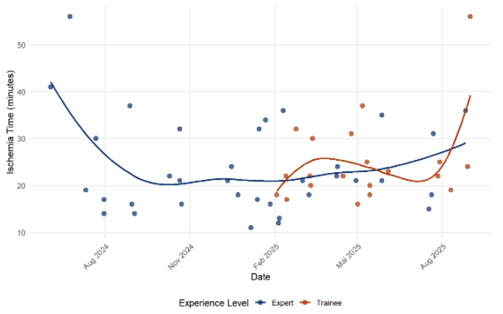

Temporal trends in ischemia time for multi-port (A-B) and single-port (C-D) robot-assisted partial nephrectomy. Individual surgeons (A, C) and pooled experience levels (B, D) are shown. Trend lines fitted using locally weighted scatterplot smoothing (LOESS). For surgeons with ≥10 cases, span=0.75; for Trainees with <10 cases, span=2.0 to prevent overfitting.

Supplementary Figure 4: Ischemia time of MP and SP surgeries stratified by surgeon and experience level

A) Multi-port robot-assisted surgery by surgeon

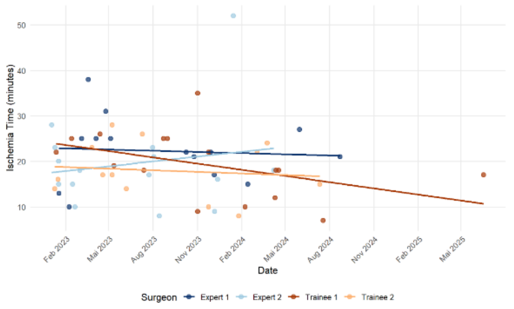

B) Multi-port robot-assisted surgery by experience level

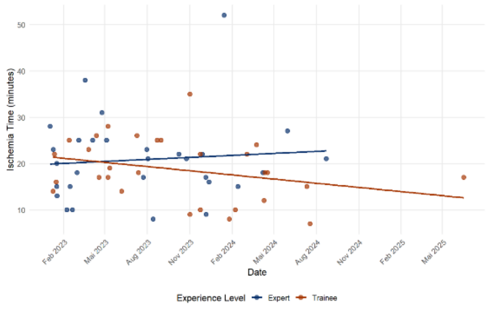

C) Single-port robot-assisted surgery by surgeon

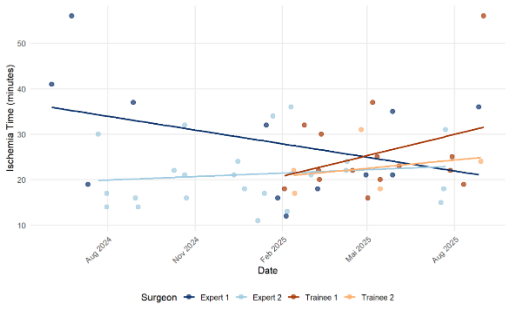

C) Single-port robot-assisted surgery by experience level

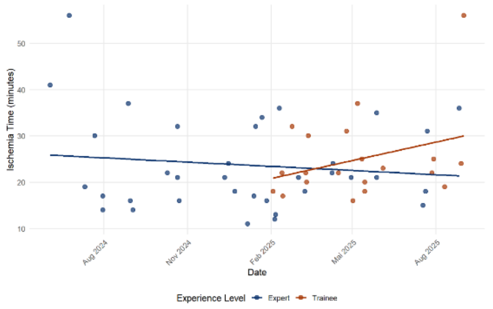

Temporal trends in ischemia time for multi-port (A-B) and single-port (C-D) robot-assisted partial nephrectomy. Individual surgeons (A, C) and pooled experience levels (B, D) are shown. Trend lines fitted using linear regression.

Supplementary Figure 5: Trifecta achievement risk over consecutive cases

A) Moving Average Analysis (10-case window)

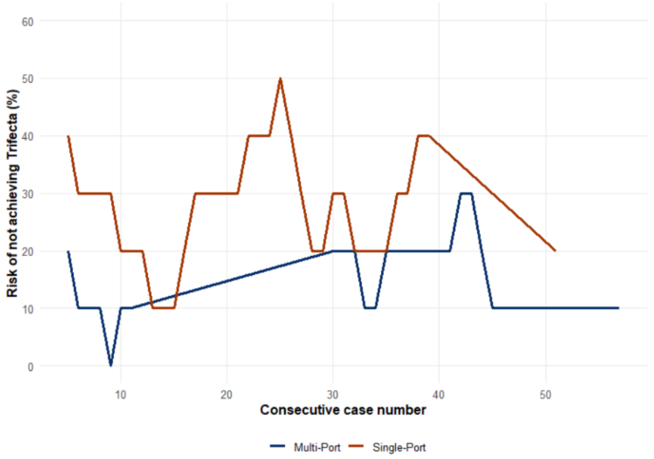

Risk of not achieving trifecta calculated as 10-case moving average centered on each consecutive case. Multi-port (blue, n=65 cases) and single-port (orange, n=62 cases). Data include all surgeons (experts and trainees combined).

C) Generalized Additive Model (GAM) with 95% Confidence Intervals

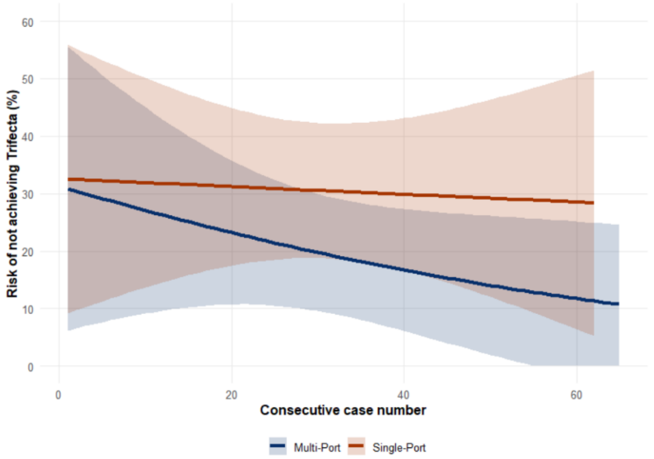

Risk estimated using binomial GAM with cubic smoothing splines (k=4). Shaded areas represent 95% confidence intervals. Multi-port (blue, n=65 cases) and single-port (orange, n=62 cases). Data include all surgeons (experts and trainees combined).

Supplementary Table 1: Detailed Charlson Comorbidity Index

Charlson Comorbidity Index Components	Overall N = 127[1]	Robot-assisted multi-port N = 65[1]	Robot-assisted single-port N = 62[1]	p-value[2]
**Myocardial infarction**				0.2
No	121 (95%)	60 (92%)	61 (98%)	
Yes	6 (4.7%)	5 (7.7%)	1 (1.6%)	
**Congestive heart failure**				0.7
No	121 (95%)	61 (94%)	60 (97%)	
Yes	6 (4.7%)	4 (6.2%)	2 (3.2%)	
**Peripheral vascular disease**				0.3
No	118 (93%)	62 (95%)	56 (90%)	
Yes	9 (7.1%)	3 (4.6%)	6 (9.7%)	
**Cerebrovascular disease**				0.7
No	120 (94%)	62 (95%)	58 (94%)	
Yes	7 (5.5%)	3 (4.6%)	4 (6.5%)	
**Dementia**				>0.9
No	126 (99%)	64 (98%)	62 (100%)	
Yes	1 (0.8%)	1 (1.5%)	0 (0%)	
**Chronic pulmonary disease**				>0.9
No	121 (95%)	62 (95%)	59 (95%)	
Yes	6 (4.7%)	3 (4.6%)	3 (4.8%)	
**Connective tissue disease**				
No	127 (100%)	65 (100%)	62 (100%)	
**Peptic ulcer disease**				>0.9
No	124 (98%)	63 (97%)	61 (98%)	
Yes	3 (2.4%)	2 (3.1%)	1 (1.6%)	
**Liver disease**				0.4
None	123 (97%)	64 (98%)	59 (95%)	
Mild	3 (2.4%)	1 (1.5%)	2 (3.2%)	
Moderate to severe	1 (0.8%)	0 (0%)	1 (1.6%)	

**Charlson Comorbidity Index Components**	**Overall** N = 127[1]	**Robot-assisted multi-port** N = 65[1]	**Robot-assisted single-port** N = 62[1]	**p-value**[2]
**Diabetes mellitus**				0.9
None or diet-controlled	115 (91%)	58 (89%)	57 (92%)	
Uncomplicated	10 (7.9%)	6 (9.2%)	4 (6.5%)	
End-organ damage	2 (1.6%)	1 (1.5%)	1 (1.6%)	
**Hemiplegia**				0.2
No	125 (98%)	65 (100%)	60 (97%)	
Yes	2 (1.6%)	0 (0%)	2 (3.2%)	
**Chronic kidney disease**				>0.9
No	113 (89%)	58 (89%)	55 (89%)	
Yes	14 (11%)	7 (11%)	7 (11%)	
**Solid tumor**				0.4
None	1 (0.8%)	0 (0%)	1 (1.6%)	
Localized	121 (95%)	61 (94%)	60 (97%)	
Metastatic	5 (3.9%)	4 (6.2%)	1 (1.6%)	
**Leukemia**				>0.9
No	126 (99%)	64 (98%)	62 (100%)	
Yes	1 (0.8%)	1 (1.5%)	0 (0%)	
**Lymphoma**				0.6
No	124 (98%)	64 (98%)	60 (97%)	
Yes	3 (2.4%)	1 (1.5%)	2 (3.2%)	

[1]n (%)

[2]Fisher's exact test

Supplementary Table 2: *Patient characteristics and outcomes of Single-Port RAPN stratified by transperitoneal vs. retroperitoneal and detailed retroperitoneal approaches*

Characteristic	Transperitoneal vs. retroperitoneal				Comparison of retroperitoneal approaches			
	**Overall SP-RAPN** (N=62)	**Transperitoneal** (N=31)	**Retroperitoneal** (LARA + SUSA + LFA) (N=31)	**p-value**	**Lateral flank access (retroperitoneal)** N = 13	**Low anterior retroperitoneal access (LARA)** N = 9	**Supine subcostal retroperitoneal access (SUSA)** N = 9	**p-value**
**Age, years**	62 (13)	64 (15)	61 (11)	0.2	59 (10)	67 (11)	57 (10)	0.2
**Gender**				0.6				0.5
female	21 (34%)	12 (39%)	9 (29%)		5 (38%)	1 (11%)	3 (33%)	
male	41 (66%)	19 (61%)	22 (71%)		8 (62%)	8 (89%)	6 (67%)	
**BMI, kg/m²**	28.3 (4.9)	27.9 (4.9)	28.6 (5.0)	0.6	30.3 (5.8)	29.1 (3.0)	25.7 (4.2)	0.2
**Charlson Comorbidity Index**	2.00 (2.00, 4.00)	2.00 (2.00, 4.00)	2.00 (2.00, 3.00)	0.3	2.00 (2.00, 3.00)	2.00 (2.00, 3.00)	2.00 (2.00, 3.00)	0.7
**ASA Classification**				**0.028**				0.2
1	1 (1.6%)	0 (0%)	1 (3.2%)		1 (8%)	0 (0%)	0 (0%)	
2	31 (50%)	11 (35%)	20 (65%)		7 (54%)	7 (78%)	6 (67%)	
3	26 (42%)	18 (58%)	8 (26%)		4 (31%)	2 (22%)	2 (22%)	
4	4 (6.5%)	2 (6.5%)	2 (6.5%)		1 (8%)	0 (0%)	1 (11%)	
**Anticoagulation**	19 (31%)	8 (26%)	11 (35%)	0.6	6 (46%)	3 (33%)	2 (22%)	0.6

Characteristic	Transperitoneal vs. retroperitoneal				Comparison of retroperitoneal approaches			
	**Overall SP-RAPN** (N=62)	**Transperitoneal** (N=31)	**Retroperitoneal** (LARA + SUSA + LFA) (N=31)	**p-value**	**Lateral flank access (retroperitoneal)** N = 13	**Low anterior retroperitoneal access (LARA)** N = 9	**Supine subcostal retroperitoneal access (SUSA)** N = 9	**p-value**
**Preoperative eGFR, mL/min/1.73 m²**	87 (71, 90)	86 (59, 90)	88 (71, 90)	0.8	89 (77, 90)	87 (78, 90)	71 (57, 90)	0.7
**Imaging modality**				0.8				0.8
CT	36 (58%)	19 (61%)	17 (55%)		7 (54%)	6 (67%)	4 (44%)	
CT and MRI	15 (24%)	8 (26%)	7 (23%)		3 (23%)	2 (22%)	2 (22%)	
MRI	10 (16%)	4 (13%)	6 (19%)		2 (15%)	1 (11%)	3 (33%)	
Szintigraphy	1 (2%)	0 (0%)	1 (3%)		1 (8%)	0 (0%)	0 (0%)	
**Tumor size, mm**	32 (22, 49)	42 (31, 50)	26 (18, 35)	**<0.001**	22 (19, 36)	31 (20, 35)	25 (16, 27)	0.002
**Lesion type**				**0.010**				0.014
Suspected cancer	51 (82%)	30 (97%)	21 (68%)		9 (69%)	6 (67%)	6 (67%)	
Bosniak Cyst ≥ 2F	4 (7%)	0 (0%)	4 (13%)		1 (8%)	1 (11%)	2 (22%)	
Suspected benign tumor	7 (11%)	1 (3%)	6 (19%)		3 (23%)	2 (22%)	1 (11%)	
**T Stage**				**0.002**				0.10
cT1a	33 (53%)	13 (42%)	20 (65%)		7 (54%)	6 (67%)	7 (78%)	
cT1b	14 (23%)	12 (39%)	2 (7%)		2 (15%)	0 (0%)	0 (0%)	

Characteristic	Transperitoneal vs. retroperitoneal				Comparison of retroperitoneal approaches			
	**Overall SP-RAPN** (N=62)	**Transperitoneal** (N=31)	**Retroperitoneal** (LARA + SUSA + LFA) (N=31)	**p-value**	**Lateral flank access (retroperitoneal)** N = 13	**Low anterior retroperitoneal access (LARA)** N = 9	**Supine subcostal retroperitoneal access (SUSA)** N = 9	**p-value**
cT2a	2 (3%)	1 (3%)	1 (3%)		1 (8%)	0 (0%)	0 (0%)	
cT2b	1 (2%)	1 (3%)	0 (0%)		0 (0%)	0 (0%)	0 (0%)	
cT3a	2 (3%)	2 (7%)	0 (0%)		0 (0%)	0 (0%)	0 (0%)	
Unknown	10 (16%)	2 (7%)	8 (26%)		3 (23%)	3 (33%)	2 (22%)	
**N Stage**								
cN0	62 (100%)	31 (100%)	31 (100%)		13 (100%)	9 (100%)	9 (100%)	
**M Stage**								
cM0	62 (100%)	31 (100%)	31 (100%)		13 (100%)	9 (100%)	9 (100%)	
**Bosniak classification**				0.11				0.023
3	1 (2%)	0 (0%)	1 (3%)		1 (8%)	0 (0%)	0 (0%)	
4	3 (5%)	0 (0%)	3 (10%)		0 (0%)	1 (11%)	2 (22%)	
**Surgery time, min**	193 (59)	213 (56)	173 (55)	**0.006**	165 (48)	159 (39)	199 (74)	
**Ischemia time, min**	21 (17, 30)	22 (18, 32)	20 (16, 25)	0.3	18 (14, 24)	19 (16, 20)	24 (21, 30)	**0.014**
**Intraoperative blood loss, ml**	90 (50, 175)	100 (100, 200)	50 (50, 100)	**0.001**	50 (50, 50)	50 (50, 50)	50 (50, 200)	0.3

Characteristic	Transperitoneal vs. retroperitoneal				Comparison of retroperitoneal approaches			
	**Overall SP-RAPN** (N=62)	**Transperitoneal** (N=31)	**Retroperitoneal** (LARA + SUSA + LFA) (N=31)	**p-value**	**Lateral flank access (retroperitoneal)** N = 13	**Low anterior retroperitoneal access (LARA)** N = 9	**Supine subcostal retroperitoneal access (SUSA)** N = 9	**p-value**
**Trifecta reached**	41 (69%)	17 (61%)	24 (77%)	0.3	11 (85%)	7 (78%)	6 (67%)	**0.002**
**Ischemia time ≤ 25 minutes**	43 (73%)	19 (68%)	24 (77%)	0.6	11 (85%)	7 (78%)	6 (67%)	0.5
**Negative resection margin**	61 (98%)	30 (97%)	31 (100%)	>0.9	13 (100%)	9 (100%)	9 (100%)	0.8
**No complication ≥ CDC Grade 3a**	61 (98%)	30 (97%)	31 (100%)	>0.9	13 (100%)	9 (100%)	9 (100%)	>0.9
**Conversion**				>0.9				>0.9
conversion to nephrectomy	1 (2%)	1 (3%)	0 (0%)		0 (0%)	0 (0%)	0 (0%)	>0.9
**Intraoperative complication**								
no	62 (100%)	31 (100%)	31 (100%)		13 (100%)	9 (100%)	9 (100%)	
**Length of stay (days)**	2.00 (2.00, 2.00)	2.00 (2.00, 3.00)	2.00 (2.00, 2.00)	**0.002**	2.00 (2.00, 2.00)	2.00 (2.00, 2.00)	2.00 (2.00, 2.00)	0.02
**Comprehensive Complication Index**	0 (0, 34)	17 (0, 34)	0 (0, 0)	>0.9	0 (0, 0)	0 (0, 0)	0 (0, 0)	>0.9
**Pathology**				0.052				0.023
Clear cell RCC	29 (47%)	17 (55%)	12 (39%)		7 (54%)	1 (11%)	4 (44%)	

Characteristic	Transperitoneal vs. retroperitoneal				Comparison of retroperitoneal approaches			
	**Overall SP-RAPN** (N=62)	**Transperitoneal** (N=31)	**Retroperitoneal** (LARA + SUSA + LFA) (N=31)	**p-value**	**Lateral flank access (retroperitoneal)** N = 13	**Low anterior retroperitoneal access (LARA)** N = 9	**Supine subcostal retroperitoneal access (SUSA)** N = 9	**p-value**
Papillary RCC	12 (19%)	3 (10%)	9 (29%)		3 (23%)	4 (44%)	2 (22%)	
Chromophobe RCC	4 (7%)	3 (10%)	1 (3%)		0 (0%)	1 (11%)	0 (0%)	
Other malignant	5 (8%)	4 (13%)	1 (3%)		0 (0%)	1 (11%)	0 (0%)	
Oncozytoma	7 (11%)	4 (13%)	3 (10%)		0 (0%)	2 (22%)	1 (11%)	
Angiomyolipoma	1 (2%)	0 (0%)	1 (3%)		0 (0%)	0 (0%)	1 (11%)	
Other benign	4 (7%)	0 (0%)	4 (13%)		3 (23%)	0 (0%)	1 (11%)	

Mean (SD) for normally distributed continuous variables; Median (Q1, Q3) for non-normally distributed continuous variables; n (%) for categorical variables.

Group comparisons performed using Student's t-test for normally distributed continuous variables, Wilcoxon rank-sum test for non-normally distributed continuous variables, and Pearson's chi-squared test or Fisher's exact test for categorical variables.

Abbreviations: ASA: American Society of Anesthesiologists; BMI: Body Mass Index; CDC: Clavien-Dindo Classification; eGFR: estimated Glomerular Filtration Rate; N: Number; RCC: Renal cell carcinoma

Supplementary Table 3: Patient characteristics and perioperative outcomes for Expert 2 Single-Port RAPN by learning phase

**Characteristic**	**Overall** (N=24)	**Before Proficiency** (Cases 1-18, N=18)	**After Proficiency** (Cases 19-24, N=6)	**p-value**
**Age, years**	64 (15)	65 (13)	61 (21)	0.8
**Gender**				0.6
female	11 (46%)	9 (50%)	2 (33%)	
male	13 (54%)	9 (50%)	4 (67%)	
**BMI, kg/m²**	26.3 (4.5)	26.9 (4.7)	24.4 (3.5)	0.3
**Charlson Comorbidity Index**	2.00 (2.00, 4.00)	2.00 (2.00, 4.00)	2.00 (2.00, 2.00)	0.4
**ASA Classification**				0.7
1	1 (4%)	1 (6%)	0 (0%)	
2	11 (46%)	7 (39%)	4 (67%)	
3	9 (38%)	7 (39%)	2 (33%)	
4	3 (13%)	3 (17%)	0 (0%)	
**Anticoagulation**	6 (25%)	6 (33%)	0 (0%)	0.3
**Preoperative eGFR, mL/min/1.73 m²**	82 (59, 90)	87 (58, 90)	73 (69, 83)	0.8
**Imaging modality**				0.053
CT	12 (50%)	9 (50%)	3 (50%)	
CT and MRI	7 (29%)	7 (39%)	0 (0%)	
MRI	5 (21%)	2 (11%)	3 (50%)	
**Tumor size, mm**	38 (22, 52)	40 (21, 54)	35 (25, 49)	0.7
**Lesion type**				0.054
Suspected cancer	21 (88%)	17 (94%)	4 (67%)	
Bosniak Cyst ≥ 2F	2 (8%)	0 (0%)	2 (33%)	
Suspected benign tumor	1 (4%)	1 (6%)	0 (0%)	
**T Stage**				0.8
cT1a	12 (50%)	9 (50%)	3 (50%)	
cT1b	5 (21%)	4 (22%)	1 (17%)	

**Characteristic**	**Overall** (N=24)	**Before Proficiency** (Cases 1-18, N=18)	**After Proficiency** (Cases 19-24, N=6)	**p-value**
cT2a	2 (8%)	2 (11%)	0 (0%)	
cT2b	0 (0%)	0 (0%)	0 (0%)	
cT3a	1 (4%)	1 (6%)	0 (0%)	
Unknown	4 (17%)	2 (11%)	2 (33%)	
**N Stage**				
cN0	24 (100%)	18 (100%)	6 (100%)	
**M Stage**				
cM0	24 (100%)	18 (100%)	6 (100%)	
**Bosniak classification**				0.054
4	2 (8%)	0 (0%)	2 (33%)	
**Surgical approach**				>0.9
Retroperitoneal	12 (50%)	9 (50%)	3 (50%)	
Transperitoneal	12 (50%)	9 (50%)	3 (50%)	
**Specific Access Technique**				0.080
Lateral flank acecess (retroperitoneal)	7 (29%)	7 (39%)	0 (0%)	
Low anterior retroperitoneal access (LARA)	2 (8%)	1 (6%)	1 (17%)	
Supine subcostal retroperitoneal access (SUSA)	3 (13%)	1 (6%)	2 (33%)	
Transperitoneal access	12 (50%)	9 (50%)	3 (50%)	
**Surgery time, min**	164 (58)	177 (57)	127 (45)	0.053
**Ischemia time, min**	20 (16, 24)	20 (16, 24)	20 (15, 24)	>0.9
**Intraoperative blood loss, ml**	50 (50, 100)	50 (50, 80)	125 (50, 300)	0.055
**Trifecta reached**	19 (79%)	14 (78%)	5 (83%)	>0.9
**Ischemia time ≤ minutes**	19 (79%)	14 (78%)	5 (83%)	>0.9
**Negative resection margin**	24 (100%)	18 (100%)	6 (100%)	

Characteristic	**Overall** (N=24)	**Before Proficiency** (Cases 1-18, N=18)	**After Proficiency** (Cases 19-24, N=6)	**p-value**
**No complication ≥ CDC Grade 3a**	24 (100%)	18 (100%)	6 (100%)	
**Conversion**				
conversion to nephrectomy	0 (0%)	0 (0%)	0 (0%)	
**Intraoperative complication**	0 (0%)	0 (0%)	0 (0%)	
**Length of stay, days**	2.00 (2.00, 2.00)	2.00 (2.00, 2.00)	2.00 (2.00, 2.00)	0.2
**Pathology**				0.4
Clear cell RCC	14 (58%)	11 (61%)	3 (50%)	
Papillary RCC	6 (25%)	5 (28%)	1 (17%)	
Other malignant	1 (4%)	1 (6%)	0 (0%)	
Oncozytoma	2 (8%)	1 (6%)	1 (17%)	
Angiomyolipom	1 (4%)	0 (0%)	1 (17%)	

Mean (SD) for normally distributed continuous variables; Median (Q1, Q3) for non-normally distributed continuous variables; n (%) for categorical variables.

Group comparisons performed using Student's t-test for normally distributed continuous variables, Wilcoxon rank-sum test for non-normally distributed continuous variables, and Pearson's chi-squared test or Fisher's exact test for categorical variables.

Abbreviations: ASA: American Society of Anesthesiologists; BMI: Body Mass Index; CDC: Clavien-Dindo Classification; eGFR: estimated Glomerular Filtration Rate; N: Number; RCC: Renal cell carcinoma
